# Barriers to care in patients with diabetes and poor glycemic control—A cross-sectional survey

**DOI:** 10.1371/journal.pone.0176135

**Published:** 2017-05-01

**Authors:** Kerry A. McBrien, Christopher Naugler, Noah Ivers, Robert G. Weaver, David Campbell, Laura Desveaux, Brenda R. Hemmelgarn, Alun L. Edwards, Nathalie Saad, David Nicholas, Braden J. Manns

**Affiliations:** 1 Department of Family Medicine, Cumming School of Medicine, University of Calgary, Calgary, Canada; 2 Department of Community Health Sciences, Cumming School of Medicine, University of Calgary, Calgary, Canada; 3 Department of Pathology and Laboratory Medicine, Cumming School of Medicine, University of Calgary, Calgary, Canada; 4 Calgary Laboratory Services, Calgary, Canada; 5 Department of Family and Community Medicine, Faculty of Medicine, University of Toronto, Toronto, Canada; 6 Women’s College Hospital, Toronto, Canada; 7 Department of Medicine, Cumming School of Medicine, University of Calgary, Calgary, Canada; 8 Diabetes, Obesity and Nutrition Strategic Clinical Network, Alberta Health Services, Canada; 9 Faculty of Social Work, University of Calgary, Calgary, Canada; International University of Health and Welfare School of Medicine, JAPAN

## Abstract

**Aims:**

To determine and quantify the prevalence of patient, provider and system level barriers to achieving diabetes care goals; and to examine whether barriers were different for people with poor glycemic control (HbA1c ≥ 10%; 86 mmol/mol) compared to fair glycemic control (7 to <8%; 53–64 mmol/mol).

**Methods:**

We administered a survey by telephone to community-dwelling patients with diabetes, to examine patient-reported barriers and facilitators to care. We compared responses in individuals with HbA1c ≥ 10% (86 mmol/mol) against those with HbA1c between 7–8% (53–64 mmol/mol). We examined associations between HbA1c group and barriers to care, adjusting for sociodemographic factors and diabetes duration.

**Results:**

The survey included 805 people with HbA1c ≥ 10% (86 mmol/mol), and 405 people with HbA1c 7–8% (53–64 mmol/mol). Participants with HbA1c ≥ 10% (86 mmol/mol) reported good access to care, however 20% of participants with HbA1c ≥ 10% (86 mmol/mol) felt that their care was not well-coordinated and 9.6% reported having an unmet health care need. In adjusted analysis, patients with HbA1c ≥10% (86 mmol/mol) were more likely to report lack of confidence and inadequate social support, compared to patients with HbA1c 7–8% (53–64 mmol/mol). They were also significantly more likely not to have drug insurance nor to have received recommended treatments because of cost.

**Conclusions:**

These results reinforce the importance of an individualized, yet multi-faceted approach. Specific attention to financial barriers seems warranted. These findings can inform the development of programs and initiatives to overcome barriers to care, and improve diabetes care and outcomes.

## Introduction

Diabetes affects more than 2.4 million Canadians [1], and is associated with high morbidity and mortality [1, 2]. Patients who do not achieve recommended clinical targets, such as glycemic control, are at higher risk of complications and incur higher health care costs [3–5]. Patients with the worst control (HbA1c ≥ 10%; 86 mmol/mol) have a 30% higher risk of myocardial infarction compared to those with HbA1c between 7 and 8% (53–64 mmol/mol) and a fourfold increased risk of microvascular complications [5].

Safe, cost-effective interventions for diabetes are available to reduce the risk of microvascular and macrovascular complications, but these treatments remain underutilized [6–13]. For instance, only 50% of Albertans with diabetes are on statin therapy, which has been shown to reduce cardiovascular risk among people with diabetes [6]. There is an urgent need to understand these care gaps to improve health outcomes in people with diabetes [14].

At the same time, patients with diabetes are burdened by complex treatment regimens including administration of medications, clinical monitoring, and dietary and lifestyle changes [15]. Patients must also make decisions about when and how to seek medical care. Optimal self-management depends on a number of factors, including health status, financial status, access to care, care experience and personal circumstances. A comprehensive understanding of patient, provider, and system-level barriers is needed to inform the development of contextually-tailored interventions to support self-management and improve outcomes for diabetes patients [16].

The purpose of this study was to determine and quantify the prevalence of patient, provider and system-level barriers to achieving diabetes care goals. We also sought to examine whether these barriers differed for people with poor glycemic control (HbA1c ≥ 10% (86 mmol/mol)) compared to fair glycemic control (7 to 8% (53–64 mmol/mol)), within four broad domains: Health status; Health care experience; Self-management; and Financial status.

## Patients and methods

We conducted a large, cross-sectional survey administered by telephone interview that asked patients about their health status, health care experience, self-management behaviours and financial barriers. The University of Calgary Conjoint Health Research Ethics Board has approved this research study.

### Participants

The target population was community-dwelling adults over 18 years living in the city of Calgary, Alberta and surrounding regions (catchment population 1.5 million), with a diagnosis of diabetes who had an HbA1c ≥ 10% (86 mmol/mol) or between 7 and 8% (53–64 mmol/mol). The sample was drawn from adult outpatient users of Calgary Laboratory Services (CLS) with valid telephone numbers, who had an outpatient HbA1c blood test between October 1, 2013 and April 30, 2014. Only patients with a prior HbA1c test ≥ 7% (53 mmol/mol) were eligible, to ensure that they had an established diagnosis of diabetes. Ordering physicians were notified by facsimile of our intent to contact their patient(s), and was given the option of contacting researchers to exclude individual patients from the survey. We included patients of family physicians, walk-in clinics and specialist clinics, to enable us to reach patients that would otherwise be inaccessible, while still providing ordering physicians the opportunity to exclude their patients if deemed inappropriate.

### Recruitment

Patients were contacted by telephone and invited to participate in a survey about diabetes care delivery in Alberta. Interviewers had no information about test results. Patients were asked to provide verbal consent to be interviewed; as the questionnaire was administered by telephone interview, it was infeasible to obtain written consent. All participants were given the option to end the survey at any time. When a participant agreed to participate in the interview, their consent was recorded as an item in the computer-assisted data capture system. The University of Calgary Conjoint Health Research Ethics Board approved this consent procedure. Individuals with gestational diabetes or who were unaware of their diabetes diagnosis were excluded. All patients were contacted between January and May 2014, within 90 days of the date of the HbA1c test to ensure that the survey data was timely and relevant. Standardized scripting was used to explain the purpose of the survey and answer questions regarding information security and privacy.

### Data collection

Trained interviewers working within the provincial health care system administered the questionnaire. Data were collected using computer-assisted telephone interviews, where participant responses were captured directly into data collection software.

### Questionnaire

The survey was organized into four domains: health status, health care experience, self-management, financial barriers; and sociodemographic information. The domains were adapted from the Barriers to Diabetes Self-Care Behaviours Model [17], and modified to include additional information on financial status, which has been identified as a barrier for some patients [18]. Within each domain, key indicators representing patient-reported barriers (Table 1) were chosen based on findings from past studies [17], and were contextualized within health care programming available for individuals with diabetes in the Calgary area. The wording of specific questions was adopted from prior Statistics Canada Health Surveys (CCHS, CSEPHC, SLCDC-DM, BCPCHC, available from www.statcan.gc.ca) and validated questionnaires where possible. Pilot testing indicated that the questions had face validity, were acceptable to patients, and could be answered within a comfortable timeframe.

**Table 1:** Key indicators of barriers and facilitators to care by survey domain.

Domain	Subdomains	Key indicators
**Health status**	**General health** **Diabetes status** **Health risks**	• General health status • Depressive symptoms
**Health care experience**	**Health care team** **Access to care** **Communication**	• Regular family doctor/usual place of care • Continuity of care with same family doctor • Accessed allied health care • Accessed specialist if recommended • Accessed specialized diabetes clinic • Accessed community programs • Communication with providers • Coordination of care across providers
**Self-management**	**Medications** **Lifestyle** **Patient activation** **Social support**	• Confidence in being able to follow through with treatments • Having knowledge to prevent further problems • Motivation to do a better job with diabetes self-management • Ability to fill out medical forms • Having adequate social support
**Financial barriers**	**Insurance status** **Ability to pay**	• Insurance to cover prescription medications? • Difficulty paying for medications • Unable to get diabetes related treatments due to cost • Able to afford healthy foods

### Analysis

Sociodemographic and clinical characteristics of the survey respondents were described by HbA1c category, using summary statistics. We summarized the prevalence of key indicators for those with poor glycemic control (HbA1c ≥ 10%; 86 mmol/mol) and those with fair glycemic control (HbA1c 7 to 8%; 53–64 mmol/mol). Modified Poisson regression [19] was used to determine the association between HbA1c group (HbA1c ≥ 10% (86 mmol/mol) or HbA1c 7 to 8% (53–64 mmol/mol)) and key indicators, adjusting for age, sex, income, ethnicity, community size and diabetes duration. Complementary data, including type of care accessed, reasons for not accessing care, and reasons for having an unmet health care need were summarized descriptively.

### Sample size

We determined sample size for statistical comparisons between HbA1c groups. We examined three key indicator variables: proportion with depression, proportion without a regular family doctor, and proportion unable to pay for medications. Based on prior work, we estimated the proportion with each of these indicators among all patients with diabetes: 12–25% with depression [17, 20, 21], 5% without a regular family doctor [22], 12% with difficulty paying for medications [22]. For a desired precision of +/- 5% in prevalence estimate, our most restrictive estimate (25% with depression) yields a minimum sample size of 384, assuming a random sample [23]. For comparisons between groups of glycemic control, assuming two independent samples and a power of 0.80 to detect a minimum detectable absolute difference in prevalence between groups of 10%, 400 patients were required in each sample [23]. To ensure the stability of our estimates and to provide some additional capacity to perform correlational analyses and comparison across sociodemographic groups we chose to aim for 800 completed interviews in patients with HbA1c ≥ 10% (86 mmol/mol), and 400 completed interviews in patients with HbA1c 7–8% (53–64 mmol/mol).

## Results

### Survey response

**Fig 1:**
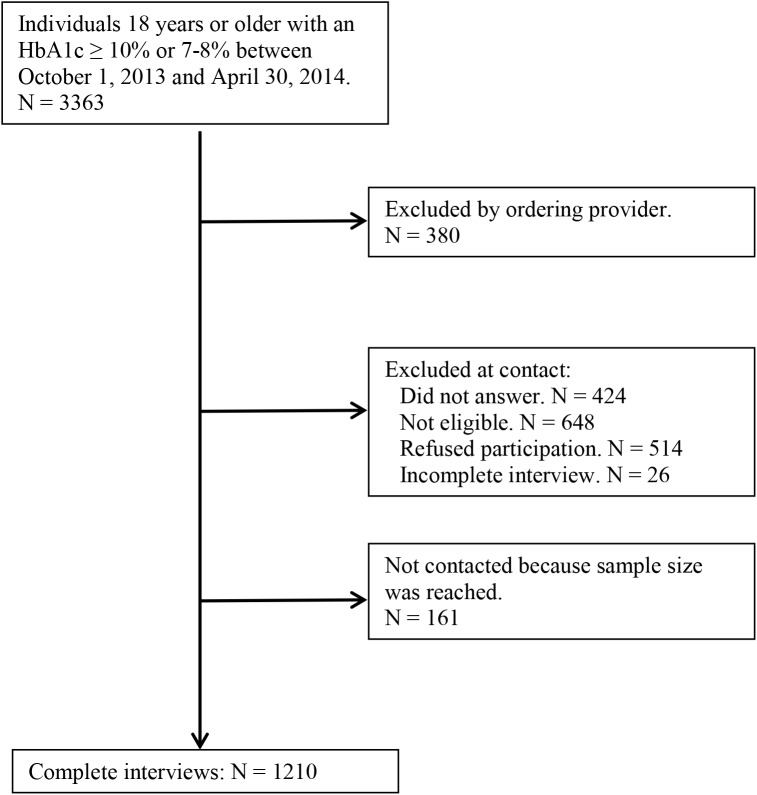
Details of participant selection.

In total, 3363 patients were identified as having HbA1c tests performed; 380 (12.7%) were excluded from contact by the ordering provider. The most common reasons for exclusion were language barrier and dementia. The average age of excluded patients was 65.2 years and 51.6% were male, compared to 60.0 years and 59.4% male for patients not excluded. Of the patients that were eligible for contact, 2822 were called. Of these, 424 did not answer, 648 were disqualified, 514 refused, and 26 did not complete the interview. Ineligibility was most commonly due to language barrier (181), telephone number not in service (154), ineligibility (101), wrong number (65), inability to communicate (55), and other. There were no differences in reasons for exclusion between the HbA1c groups. We thus completed 1210 surveys, with a 44% response rate overall, and a 71% response rate in those who were contacted and qualified to participate (Fig 1). There was no significant difference in response rate by sex or HbA1c group, though we did observe lower response rates in those aged ≥75.

### Patient characteristics

Table 2 summarizes patient characteristics by HbA1c group. Patients with HbA1c ≥ 10% (86 mmol/mol) had an average age of 57.1 years, and 60% were male. Most were high school educated (88%), and were employed, retired, or in school (86.2%). Household income varied, with 13.8% having an annual household income of <$20,000. Two-thirds were born in Canada, 71% were white, and over 75% were city dwellers. Compared to the group with HbA1c 7–8% (53–64 mmol/mol), individuals with HbA1c ≥ 10% (86 mmol/mol) were younger and fewer were retired (23.2% vs. 47.3%). Average income was higher in the HbA1c ≥ 10% (86 mmol/mol) group, although this may reflect fewer retirees in this group. There was also a higher proportion of rural/small community dwellers in the HbA1c ≥ 10% (86 mmol/mol) group (15.8% vs. 4.2%).

**Table 2:** Patient characteristics summarized by HbA1c group [n (%)].

	HbA1c ≥ 10% N = 805	HbA1c 7–8% N = 405	p-value
***Sociodemographics***			
Age in years, mean (s.d.)	57.1 (12.5)	64.5 (11.8)	<0.001
*Age group*			<0.001
<50	200 (24.8)	37 (9.1)	
50–64	389 (48.3)	142 (35.1)	
65–74	150 (18.6)	152 (37.5)	
75+	66 (8.2)	74 (18.3)	
Male	60.4	58.8	0.59
*Education–highest level achieved*			0.73
Less than high school	95 (12.0)	47 (11.9)	
High school	227 (28.6)	104 (26.3)	
Post-secondary but no bachelor’s degree	279 (35.2)	151 (38.2)	
Bachelor’s degree or higher	192 (24.2)	93 (23.5)	
*Employment*			<0.001
Employed FT/PT/self-employed	477 (59.5)	160 (39.6)	
Unemployed, looking for work	24 (3.0)	6 (1.5)	
Unable to work–sickness or disability	87 (10.8)	35 (8.7)	
Retired	186 (23.2)	191 (47.3)	
Other (at school, full-time home-maker)	28 (3.5)	12 (3.0)	
*Household income*			0.03
< $20,000	99 (13.8)	46 (12.9)	
$20,000 to < $50,000	200 (27.9)	132 (37.1)	
$50,000 to < $100,000	235 (32.7)	95 (26.7)	
$100,000+	184 (25.6)	83 (23.3)	
Unreported	87 (10.8)	49 (12.1)	
*Marital status*			0.67
Married/common-law	539 (67.5)	275 (68.8)	
Widowed/separated/divorced/never married	259 (32.5)	125 (31.2)	
*Born in Canada*			0.86
Yes	532 (66.2)	269 (66.8)	
No	271 (33.8)	134 (33.2)	
*Racial/cultural origin*			0.13
White	564 (71.0)	306 (76.5)	
Aboriginal	33 (4.2)	12 (3.0)	
Other	197 (24.8)	82 (20.5)	
*Community size*			<0.001
Large population centre (100,000+)	615 (76.8)	368 (90.8)	
Medium population centre (30,000–99,999)	59 (7.4)	20 (5.0)	
Small population centre (1,000–29,999)	82 (10.2)	11 (2.7)	
Rural area	45 (5.6)	6 (1.5)	
***Diabetes treatment***			
*Duration of diabetes*			0.62
< 1 year	34 (4.2)	22 (5.5)	
1 to 5 years	202 (25.1)	91 (22.6)	
6 to 10 years	205 (25.5)	108 (26.8)	
> 10 years	363 (45.2)	182 (45.2)	
*Number of prescribed medications*			0.18
0–2	139 (17.3)	68 (16.9)	
3–4	250 (31.2)	103 (25.6)	
5–9	318 (39.6)	182 (45.3)	
10+	95 (11.8)	49 (12.2)	
Insulin	493 (61.2)	166 (41.2)	<0.001
Other diabetic agents	603 (75.3)	294 (73.3)	0.46
Anti-hypertensive agents	538 (67.1)	302 (75.1)	0.004
Cholesterol-lowering agents	501 (62.8)	292 (73.0)	<0.001
ASA	371 (46.2)	242 (59.8)	<0.001
***Health status***			
*General health status*			0.015
Excellent/very good	199 (24.9)	126 (31.3)	
Good	351 (43.9)	171 (42.4)	
Fair/poor	249 (31.2)	106 (26.3)	
Hypertension	544(67.9)	296 (73.4)	0.048
Retinopathy	133 (17.1)	59 (15.4)	0.46
Heart disease	164 (20.5)	99 (24.7)	0.10
Neuropathy	182 (23.6)	83 (21.7)	0.48
*Obesity*			0.002
Non-obese (BMI < 30)	380 (48.5)	223 (57.3)	
Obese (BMI 30–40)	323 (41.2)	139 (35.7)	
Morbidly obese (BMI > 40)	81 (10.3)	27 (6.9)	
Depressive symptoms	338 (42.2)	141 (34.8)	0.013
Current smoker	102 (12.8)	40 (9.9)	0.15

### Health status

Nearly half of participants reported having diabetes for over 10 years, and over half were taking five or more medications, with no difference between groups. 61.2% of patients with HbA1c ≥ 10% (86 mmol/mol) were on insulin, which was significantly greater than those with HbA1c 7–8% (53–64 mmol/mol) (i.e., 41.2%). Conversely, the proportion of participants in the HbA1c ≥ 10% (86 mmol/mol) group that were on anti-hypertensive (67.1%) and/or cardioprotective medications (62.8% for lowering cholesterol and 46.2% for ASA therapy) was significantly lower than in the HbA1c 7–8% (53–64 mmol/mol) group. Patients with HbA1c ≥10% (86 mmol/mol) were significantly more likely to report fair or poor health status (31.2% vs. 26.3%), obesity (51.5% vs. 42.8%) and depressive symptoms (42.2% vs. 34.8%).

### Health care experience

Nearly all patients in our survey (98%) reported having a regular family doctor or usual place of care, and 90% and 93% of those with HbA1c ≥10% (86 mmol/mol) and HbA1c 7–8% (53–64 mmol/mol), respectively, reported seeing the same doctor most or all of the time (Table 3). Patients with HbA1c ≥10% (86 mmol/mol) saw their family doctor more frequently than those with HbA1c 7–8% (53–64 mmol/mol), with 20% of patients reporting more than six visits in the prior 12 months. Patients with HbA1c ≥10% (86 mmol/mol) were also more likely to have seen an allied health care practitioner (77.5% vs. 69.6%) and were more likely to have been referred to a specialist (47.1% vs. 36.1%). When asked specifically about encounters with other health care professionals, pharmacists were the most prevalent (56%), followed by diabetes nurse (40%), dietician (35%), social worker (6.7%) and mental health worker (6.7%). Patients reported accessing a range of community programs, including group education, group exercise, and support groups, with no significant differences between groups. Finally, 9.6% of patients with HbA1c ≥ 10% (86 mmol/mol) reported that they had an unmet health care need related to diabetes in the last 12 months, compared with 4.8% of those with HbA1c 7–8% (53–64 mmol/mol). The most commonly cited reasons for having an unmet health care need were unsatisfactory care and/or communication, long wait times and cost.

**Table 3:** Patient responses by survey domain.

	HbA1c ≥ 10%	HbA1c 7–8%	p-value
***Health care experience***			
Regular family doctor/usual place of primary care	785 (97.5)	398 (98.3)	0.40
Continuity with primary care physician	713 (89.8)	372 (93.0)	0.07
*# of visits with family doctor in last 12 months*			0.001
0	30 (3.7)	23 (5.7)	
1	49 (6.1)	42 (10.4)	
2–6	559 (69.7)	276 (68.3)	
>6	164 (20.5)	63 (15.6)	
Use of allied health care in past 12 months	624 (77.5)	281 (69.6)	0.003
Referred to a specialist for diabetes	379 (47.3)	146 (36.1)	<0.001
Specialist care in past 12 months (if referred, n = 525)	270 (71.2)	111 (76.0)	0.27
Contact with diabetes clinic in past 12 months	167 (20.8)	62 (15.4)	0.023
Use of community programs past 12 months	143 (17.8)	60 (14.9)	0.20
Unmet health care need in last 12 months	75 (9.6%)	19 (4.8%)	0.005
Suboptimal provider communication	137 (17.8)	64 (16.4)	0.53
Suboptimal coordination of care	143 (20.1)	48 (14.2)	0.02
***Self-management***			
*Patient activation*			
Not confident can follow through	32 (4.0)	5 (1.2)	0.008
Don’t know how to prevent further problems	84 (10.6)	27 (6.9)	0.038
*Motivated to do a better job managing diabetes*			0.001
Strongly agree/agree	596 (74.5)	270 (67.0)	
Disagree/strongly disagree	46 (5.8)	16 (4.0)	
N/A (already doing a good job)	158 (19.8)	117 (29.0)	
Lacking confidence in filling out medical forms	312 (39.8)	142 (36.1)	0.22
Not enough information re managing diabetes	143 (18.0)	66 (16.4)	0.49
*Enough social support re diabetes*			0.01
Always/often	455 (59.9)	252 (67.4)	
Sometimes	108 (14.2)	47 (12.6)	
Rarely/never	197 (25.9)	75 (20.0)	
***Financial barriers***			
No drug insurance	108 (13.4)	36 (8.9)	0.02
Difficulty paying for drugs/equip/services (always/often/sometimes)	233 (29.4)	95 (23.6)	0.03
Did not get needed treatments because of cost (always/often/sometimes)	119 (15.0)	36 (9.0)	0.003
Unable to afford healthy foods (always/often/sometimes)	164 (20.8)	56 (14.1)	0.005

Patients who did not have a family doctor were asked to provide a reason, with the most common response being that their family doctor had retired or moved (51.6%). Of patients who were referred to a specialist physician but did not see one (24% for HbA1c 7–8% (53–64 mmol/mol) and 29% for HbA1c ≥ 10% (86 mmol/mol)), the most common reasons for not going were that it ended up not being necessary, long wait times and lack of time. 17.8% of participants with HbA1c ≥ 10% (86 mmol/mol) felt that provider communication was suboptimal, with no difference between groups; patients with HbA1c ≥10% (86 mmol/mol) were significantly more likely to report suboptimal provider coordination of care (20.1% vs. 14.2%).

### Self-management and financial barriers

Despite poor glycemic control, individuals with HbA1c ≥ 10% (86 mmol/mol) were more likely to be motivated (74.5% vs. 67.0%) than those with HbA1c 7–8% (53–64 mmol/mol), but also more likely to report lack of confidence in being able to follow through with their treatment plan (4.0% vs. 1.2%) and/or lack of skills to prevent disease related-complications (10.6% vs. 6.9%). Individuals with HbA1c ≥ 10% (86 mmol/mol) were less likely to have good social support (59.9% vs. 67.4%), and were more likely to have financial barriers to care, compared with the HbA1c 7–8% (53–64 mmol/mol) group. Specifically, more individuals with HbA1c ≥ 10% (86 mmol/mol) did not have drug insurance (13.4% vs. 8.9%), and reported not being able to access medications and/or medical supplies due to cost (15.0% vs. 9.0%), or to afford healthy foods (20.8% vs. 14.1%) (Table 3).

### Adjusted comparisons between HbA1c groups for key indicators

**Fig 2:**
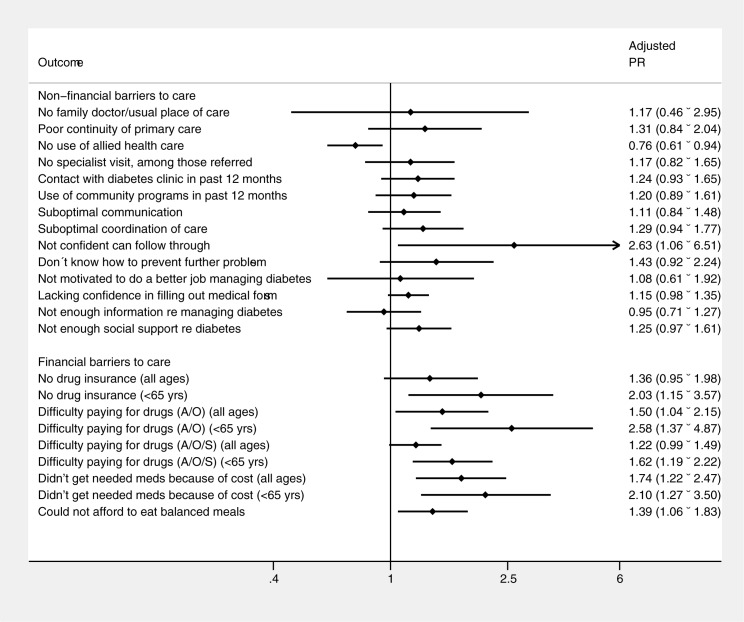
Adjusted prevalence ratios for barriers to care in participants with HbA1c ≥ 10% compared with participants with HbA1c 7–8%.

We examined the statistical association between HbA1c group and the prevalence of key indicators of barriers and facilitators to care, adjusting for sociodemographic factors and duration of diabetes (Fig 2). After adjustment for covariates, we found that participants with HbA1c ≥ 10% (86 mmol/mol) remained more likely to report a lack of confidence (PRR 2.36, 95% CI 1.06–6.51). We found significant associations between having an HbA1c ≥ 10% (86 mmol/mol) and financial barriers, with a stronger association among those under 65 (i.e., a population of individuals who have to pay a premium to access government-sponsored drug insurance). Participants with HbA1c ≥10% (86 mmol/mol) were more likely to report not having drug insurance (PRR 2.03, 95% CI 1.15–3.57, for those under 65), not getting needed medications because of cost (PRR 1.74, 95% CI 1.22–2.47, for all participants) and difficulty affording balanced meals (PRR 1.39, 95% CI 1.06–1.83, for all participants).

## Conclusions

In this population-based survey of individuals with diabetes, we examined factors associated with diabetes care and potential barriers to care across several domains, and compared responses from patients with poor glycemic control to those with fair glycemic control. We found that, in general, people with HbA1c ≥ 10% (86 mmol/mol) were younger but in worse health than those with HbA1c 7–8% (53–64 mmol/mol). We found few differences in access to care between groups but noted that patients with HbA1c ≥ 10% (86 mmol/mol) were more likely to struggle with confidence and appeared to have fewer social supports. Financial barriers were significantly more common in patients with HbA1c ≥ 10% (86 mmol/mol), and these were multifactorial. Given the cross-sectional nature of this survey, it is not possible to comment on causation. However, the data may be used to generate hypotheses regarding targeted strategies to improve diabetes management and outcomes in this population.

Disparities in glycemic control in this population are unlikely to be related to lack of access to care. Those with HbA1c ≥ 10% (86 mmol/mol) reported seeing their family physician and other members of the health care team (allied health practitioners and specialists) more often than those with HbA1c 7–8% (53–64 mmol/mol). However, nearly one quarter of patients with HbA1c ≥ 10% (86 mmol/mol) had not seen an allied health care practitioner for diabetes. Given the evidence for improvement in disease control with the use of interdisciplinary teams [14], this represents a potential opportunity to improve care. Further involvement of care teams may also improve perceived coordination of care [14], which was more commonly reported as suboptimal by patients with poor glycemic control.

Low motivation to improve self-management skills does not seem to be a common barrier for patients with poor or adequate glycemic control. However, only 18% of individuals with HbA1c ≥ 10% (86 mmol/mol) felt they needed more information, which may reflect low confidence in being able to put information into use. Low levels of social support in this group may be a factor, as lack of social support has been linked to low adherence and low self-efficacy [24, 25]. Similarly, depressive symptoms were more common in the HbA1c ≥ 10% (86 mmol/mol) group, and depression has been associated with low adherence to both lifestyle changes and medications [17, 26, 27]. Treatment strategies that aim to increase patient empowerment, and enhance social support via peer support may be useful in addressing these barriers. Specifically, personalized care planning has been shown to improve self-care and lead to improvements in glycemic control [28].

We found that patients with HbA1c ≥ 10% (86 mmol/mol) were more likely to report financial barriers, including not having drug insurance, not getting needed treatments because of cost, and not being able to afford a healthy diet. These results align with studies from other jurisdictions noting an association between cost and glycemic control [17, 29, 30]. Financial barriers increase the risk of non-adherence and are associated with higher utilization of acute care services among chronic disease patients [18, 31]. Our finding may be due to the fact that patients with HbA1c ≥ 10% (86 mmol/mol) were more likely to be under 65 years than those with HbA1c 7–8% (53–64 mmol/mol); financial barriers to obtaining requisite treatments were less common in patients over 65 years, which is the age at which Alberta residents receive universal medication insurance. Access to affordable, healthy food is essential for diabetes self-management and individuals with food insecurity are limited in their ability to prepare appropriate foods and maintain adequate meal spacing [32]. Food insecurity and medication non-adherence often coexist [33, 34]. Increasing access to universal medication insurance and nutritional assistance programs are potential strategies for overcoming financial barriers [34, 35]. Patient navigator programs, in which trained personnel assist patients in accessing available resources, are another potential approach to mitigating financial barriers to care [36, 37].

Our study has several strengths, including large sample size, comparison of responses between HbA1c groups, breadth of the survey questions, and excellent response rate. Some limitations are also worth considering. First, our inclusion criteria may have led to underrepresentation of certain ethnic groups due to language restrictions, and limited our ability to reach vulnerable patients who are not regularly accessing care and are less likely to get routine blood work done. Second, patient responses may reflect the specific resources and services available in the region, and potentially limit the generalizability of our findings. Third, we did not capture data for some lifestyle elements (e.g., sleep, description of employment, physical activity and leisure activities) that may influence diabetes self-care. Finally, the cross-sectional nature of the survey prevents us from drawing conclusions regarding causality.

In conclusion, we found that patients with poor glycemic control face multiple barriers to care, and though similar barriers were reported by those with HbA1c 7–8% (53–64 mmol/mol), significantly more individuals with poor glycemic control reported financial barriers. Given the heterogeneity of barriers across individuals, tailored strategies to overcome modifiable barriers, in light of the significant financial burden of diabetes, are urgently needed.
